# Use of bisphosphonates in chronic kidney disease is associated with cardiovascular death 

**DOI:** 10.5414/CN111428

**Published:** 2024-11-14

**Authors:** Kathleen A. Borghoff, Agnes E. Ounda, Melissa L. Swee, Saket Girotra, Amal A. Shibli-Rahhal, Patrick Ten Eyck, Diana I. Jalal, Anna J. Jovanovich

**Affiliations:** 1 University of Nebraska Medical Center, Omaha, NE,; 2 UT Health Tyler, Tyler, TX,; 3 University of Iowa, Iowa City, IA,; 4 University of Texas Southwestern, Dallas, TX,; 5 Institute for Clinical and Translational Science, University of Iowa,; 6 Iowa City VA Medical Center, Iowa City, IA , and; 7 Bozeman Health, Bozeman, MT, USA

**Keywords:** bisphosphonates, cardiovascular disease, chronic kidney disease

## Abstract

Background and objectives: Chronic kidney disease (CKD) is associated with increased cardiovascular risk, which may be mediated by vascular calcification. Based on evidence that bisphosphonates inhibit vascular calcification, we hypothesized use of bisphosphonates in CKD would be associated with lower incident cardiovascular disease (CVD), CVD-related mortality, and all-cause mortality. Materials and methods: This was a longitudinal observational study including 2,593 Framingham Offspring participants. We used propensity score-adjusted Cox regression models to determine the association between bisphosphonate use and outcomes: time to incident CVD, time to CVD-related mortality, and time to all-cause mortality. The data were stratified by presence or absence of CKD, defined as estimated glomerular filtration rate < 60 mL/min/1.73m^2^. The propensity score included age, sex, hypertension, smoking status, diabetes, total cholesterol, high-density lipoprotein, and self-reported history of fracture. Results: In unadjusted and propensity score-adjusted analyses, those with CKD using bisphosphonates had a trend toward increased incident CVD risk (adjusted hazard ratio (HR) 1.66 (95% CI, 93 – 2.97)) compared to those with CKD not using bisphosphonates. Those with CKD using bisphosphonates also had increased risk of cardiovascular mortality in the propensity score-adjusted model (adjusted HR 2.20 (95% CI, 1.12 – 4.32)). There was no significant association between bisphosphonate use and all-cause mortality in participants with CKD. Among individuals without CKD, bisphosphonate use was significantly associated with an increase in all-cause mortality in the propensity-score adjusted analysis (adjusted HR 1.59 (95% CI, 1.27 – 1.98)). However, there was no significant association between bisphosphonate use and incident CVD events (adjusted HR 0.85 95% CI, 0.63 – 1.16) or CVD-related death (adjusted HR 0.70 (95% CI 0.36 – 1.37) in those without CKD. Conclusion: Contrary to our hypothesis, bisphosphonate use was associated with a trend toward increased incident CVD and a two-fold higher risk of CVD mortality in CKD.

## Introduction 

Over 19 million Americans suffer from chronic kidney disease (CKD) [1]. CKD is associated with increased risk of cardiovascular disease (CVD), and CVD is the leading cause of death in those with CKD [2, 3]. This increased CVD risk stems in part from CKD-mineral bone disorder (CKD-MBD) and the associated biochemical mineral abnormalities, bone changes and fracture, and vascular calcification [4]. 

Vascular calcification is a complex and highly regulated process whereby hydroxyapatite crystals deposit in the intimal or medial layer of arteries [5]. Mineral bone biochemical abnormalities, including hyperparathyroidism, vitamin D deficiency, and fibroblast growth factor 23 excess result in the dysregulation of phosphorus and calcium metabolism. This plays a role in the initiation and progression of vascular calcification in patients with kidney disease leading to an increased risk of CVD and death [5, 6]. 

Bisphosphonates are the most-commonly used drugs in the general population to treat osteoporosis and to prevent bone fractures [7]. They act by inhibiting osteoclast activity and stimulating osteoclast apoptosis [7, 8], thus decreasing bone turnover and allowing a longer bone mineralization phase with increased calcium deposition in the bone. While the pathogenesis of bone disease in CKD is different from the pathogenesis of osteoporosis in the general population, bisphosphonates are nonetheless used to prevent fracture in individuals with mild to moderate CKD [9, 10, 11, 12]. 

Bisphosphonates prevent or reduce vascular calcification in animal studies [5, 13] and are associated with reduced arterial calcification in humans [8]. In non-CKD cohorts, secondary analyses of large clinical trials suggest bisphosphonates may be protective against CVD [14]. However, no study to date has evaluated if bisphosphonates are associated with a lower risk of CVD in those with CKD. Here, we hypothesized that bisphosphonate use would reduce the risk of CVD events and mortality among individuals with and without CKD. 

## Materials and methods 

### Participants 

The Framingham Offspring study was utilized for this analysis [15]. Briefly, the Framingham Offspring study was a community-based, observational, prospective investigation of risk factors for CVD. It enrolled 5,124 adults who were children of the original Framingham Heart Study participants, and their spouses. Enrollment began in 1971, and participants were formally evaluated every 4 – 8 years throughout their lifespan. At each examination cycle, a focused medical history and physical exam was undertaken. Additionally, an electrocardiogram was performed, and blood and urine samples were collected to gather information on cardiovascular risk. 

We included 2,593 Framingham Offspring participants evaluated at examination cycle 8 (2005 – 2008) or 9 (2011 – 2014). For our analysis, examination cycle 8 was considered baseline. We evaluated the medication list collected at examination cycle 9, and if bisphosphonates were started in the interim, examination cycle 9 was used as the baseline visit for those participants instead of examination cycle 8. 

The Framingham Offspring study was conducted in accordance with the Declaration of Helsinki. All study participants provided written informed consent, and the study protocol was approved by the Boston University Medical Center Review Board. 

### Predictor variable 

The predictor variable for our analysis was bisphosphonate use at examination cycle 8 or 9 and included the following medications: etidronate, alendronate, risedronate, zoledronate, ibandronate, and pamidronate. 

### Outcomes 

The outcome variables for our analysis included: (a) time to incident CVD among participants with no prior history of CVD (n = 2,128), (b) time to CVD-related mortality, and (c) time to all-cause mortality. CVD was defined as coronary heart disease, congestive heart failure and/or stroke/transient ischemic attack per the Framingham Heart Study standardized criteria [16]. 

### Statistical analysis 

Normally distributed continuous baseline variables are described as mean + standard deviation (SD), while baseline variables with skewed distributions are described with median and interquartile range (IQR). Baseline categorical variables are described with frequency and percentage. Participants were stratified by presence or absence of CKD, defined as CKD-EPI estimated glomerular filtration rate (eGFR) < 60 mL/min/1.73m^2^. We compared baseline measures between those with bisphosphonate use and those without bisphosphonate use using two-sample t- or Wilcoxon rank sum tests for continuous variables and Pearson’s χ^2^- or Fisher’s exact test for categorical variables. We used Cox proportional hazard models to determine the relationship between bisphosphonate use and the time to each outcome. Propensity scores were calculated separately for participants with CKD and participants without CKD. We employed logistic regression to calculate estimates of the conditional probability of bisphosphonate use in the CKD and non-CKD groups (c-statistics are 0.791 and 0.785, respectively). The estimated probabilities serve as propensity scores and were used as inverse-probability of treatment weights in the Cox regression models. These adjusted models are compared to their unadjusted analogues, which do not employ any propensity score correction. The variables used for the development of the propensity score were selected based on their clinical importance and their availability in the Framingham Offspring dataset and included: clinical indications for bisphosphonate use (self-reported history of fracture, use of systemic corticosteroids, and malignancy), age, sex, hypertension, smoking status, diabetes, total cholesterol, and high-density lipoprotein (HDL). A p-value of < 0.05 was used to determine statistical significance. All statistical analyses were performed using SAS for Windows, version 9.4 (SAS Institute Inc., Cary, NC, USA). 

## Results 

### Baseline characteristics 

A total of 2,593 individuals were included in the analysis, and 317 used bisphosphonates. The median (IQR) follow-up time for incident CVD was 3.94 (1.73 – 6.05) years, CVD-related mortality was 4.27 (2.38 – 6.22) years, and all-cause mortality was 5.13 (2.91 – 6.79) years. The mean ± SD age was 70 ± 9 years, and 10% were male. The prevalence of CKD (defined as eGFR < 60 mL/min/1.73m^2^) among those who used bisphosphonates and who did not use bisphosphonates was similar, 10% and 11%, respectively. Baseline characteristics of participants by CKD status and use of bisphosphonates are summarized in Table 1. Of the 2,593 participants, 284 (12%) had CKD. The group without CKD was younger than the CKD group. Within the non-CKD group, those who used bisphosphonates were older than those who did not use bisphosphonates. In the CKD group, age was similar between those who used bisphosphonates and those who did not. In both the non-CKD and CKD group, there were fewer men and lower prevalence of diabetes among those who used bisphosphonates. History of fracture was higher in the CKD group compared to the non-CKD group. 

### The association between bisphosphonate use and incident CVD 

A total of 2,128 participants did not have prevalent CVD and were included in the analysis evaluating the association between bisphosphonate use and time to incident CVD (Table 2). In the unadjusted analysis, those with CKD who used bisphosphonates had a non-significant increase in incident CVD (hazard ratio (HR) 1.72 (95% CI, 0.89 – 3.33)) compared to those with CKD who did not use bisphosphonates. In the propensity score-adjusted model, the association between bisphosphonate use in those with CKD and incident CVD was only slightly attenuated and remained non-significant. Similarly, there was no significant association between bisphosphonate use and incident CVD in those without CKD (propensity score-adjusted HR 0.85 (95% CI, 0.63 – 1.16)). 

### The association between bisphosphonate use and mortality 

In those with CKD, there was a significant association between bisphosphonate use and increased CVD-related mortality in the propensity score-adjusted model (HR 2.20 (95% CI 1.12 – 4.32)). Conversely, among participants without CKD who used bisphosphonates, we found no significant association with CVD-related mortality (adjusted HR 0.70 (95% CI 0.36 – 1.37)) (Table 2). 

Finally, we explored the relationship between bisphosphonate use and time to all-cause mortality (Table 2). There was no significant association between bisphosphonate use among those with CKD and all-cause mortality in the unadjusted analysis (HR 1.05 (95% CI, 0.66 – 1.67)). While HR increased in the propensity score-adjusted analysis, it did not quite meet statistical significance (HR 1.42 (95% CI, 0.90 – 2.22)). Among those without CKD, there was no association between bisphosphonate use and all-cause mortality in the unadjusted model (HR 1.14 (95% CI, 0.88 – 1.48)); however, after propensity score adjustment, bisphosphonate use was significantly associated with an increase in all-cause mortality (HR 1.59 (95% CI, 1.27 – 1.98)). 

## Discussion 

In this analysis of data from the Framingham Offspring study, we found that bisphosphonate use was associated with a trend toward increased risk of incident CVD and a greater than two-fold higher risk of cardiovascular mortality in individuals with CKD independent of propensity score adjustment, which included age, sex, hypertension, smoking status, diabetes, total cholesterol, HDL, and factors related to bisphosphonate use: self-reported fracture, steroid use, and malignancy. While there was a trend toward an increase in all-cause mortality among those with CKD who used bisphosphonates, the HR did not reach statistical significance. Bisphosphonate use in subjects without CKD was only associated with an increase in all-cause mortality and was not associated with incident CVD or CVD-related mortality. These data suggest differential effects of bisphosphonate use on the cardiovascular system according to the presence or absence of CKD among community-dwelling individuals. 

There is a strong association between vascular calcification and CVD. Considering that some animal and clinical studies have shown a decrease in vascular calcification with bisphosphonate use [7, 8, 13, 14], we expected to see a protective effect of bisphosphonate use on CVD events and mortality including in those with CKD. However, one study by Nik and colleagues found that bisphosphonate administration late in the course of atherosclerosis alters mineral morphology in a manner that may destabilize the atherosclerotic plaque (it leads to smaller average mineral size) [17]. Indirectly, bisphosphonates may aggravate adynamic bone disease in CKD, increasing the risk of vascular calcification further [18, 19, 20]. In addition, patients using bisphosphonates often take additional calcium supplementation [21], which in patients with CKD can result in a positive calcium balance and may result in vascular calcification [22]. 

While some animal experiments and small studies of dialysis patients suggest that the first-generation bisphosphonate, etidronate, reduces and/or slows progression of coronary artery calcification [23, 24], several more recent studies indicate that later-generation bisphosphonates may not confer such vascular benefits. In a randomized controlled trial in 51 patients with eGFR 20 – 60 mL/min, there was no difference in progression of aortic calcification after 18 months of treatment with alendronate compared to controls [25]. Likewise, bisphosphonates were not associated with cardiovascular event rates among women with CKD in a retrospective analysis [26], and in a post hoc analysis of the HORIZON trial, treatment with alendronate did not change abdominal aortic calcification in post-menopausal women [27]. In the analysis reported here, the majority of participants were treated with alendronate, a later-generation bisphosphonate. Our results not only challenge the notion that bisphosphonates confer cardiovascular benefit in CKD but suggest their use may increase cardiovascular risk. 

There are no randomized control trials looking at bisphosphonate use and cardiovascular and mortality outcomes in CKD patients, though some have examined their use in transplant patients. There are a few retrospective studies that analyze bisphosphonates in CKD, including one by Hartle and colleagues [26]. Their study saw an association between bisphosphonate use and lower risk of mortality in women with CKD [26]. Of note, their data showed a trend towards bisphosphonate use and higher risk of cardiovascular outcomes (adjust HR 1.14; 95% CI 0.94 – 1.39, p = 0.2) [26]. So, while they identified an association with bisphosphonate use and lower mortality risk, this was not attributed to reduced cardiovascular events. 

There are also no large randomized controlled trials evaluating the safety and efficacy of bisphosphonates among individuals with CKD. A few secondary analyses of clinical trials that included some CKD patients suggest bisphosphonates are safe in patients with CKD [10, 11, 12]. In a pooled analysis of nine clinical trials, there was no difference in overall adverse events or kidney function-related adverse events in 8,996 postmenopausal women with reduced kidney function (Cockcroft-Gault creatinine clearance (CrCl) 30 – 80 mL/min) taking risedronate compared placebo [10]. In another analysis of 581 women with osteoporosis treated with alendronate or placebo, there was no difference in severe or kidney-related adverse events among participants with severe kidney impairment (Cockcroft-Gault CrCl < 45 mL/min) compared to those with CrCl ≥ 45 mL/min [11]. However, none of these studies were powered to investigate cardiovascular outcomes as a primary outcome. 

Bisphosphonate use in participants without CKD was associated with an increase in all-cause mortality but not incident CVD or CVD-related mortality, suggesting causes of death other than CVD in this group. This finding is likely due to the increased risk of mortality associated with osteoporosis especially osteoporotic fractures of the hip and spine [28]. 

Our study has several limitations. A principal limitation of this study is the small number of event rates and participants that used bisphosphonates, especially in the CKD group. This was an observational cohort study, and as such, we were not able to establish causality. Of note, we were unable to discern the underlying clinical concern that would have led to prescribing these drugs from the database. The majority of the participants were older and female, and thus our findings may not be applicable to males and younger populations. Lastly, although a propensity score was utilized to adjust for confounding variables, some unmeasured confounders may still exist, such as dual energy X-ray absorptiometry (DXA) data. For example, bisphosphonates are typically prescribed to patients with low bone mineral density on DXA [29], and low bone density itself is associated with greater vascular calcification in CKD [30]. Therefore, the association between bisphosphonate use and adverse cardiovascular outcomes in participants with CKD might also reflect a higher baseline risk in this population that our analyses did not adjust for. Additionally, we were unable to distinguish between medial and intimal calcification, and there may be unaccounted for differential effects of bisphosphonates on calcification – there is evidence that bisphosphonates may increase calcification in vitro while other studies suggest the opposite. Despite these limitations, our analysis has several strengths, including a population with well-documented adjudicated cardiovascular outcomes, a long follow-up period, the inclusion of men, and the development and inclusion of a propensity score to adjust for confounding variables including factors related to bisphosphonate use. 

In conclusion, bisphosphonate use in individuals with CKD was associated with a trend toward increased risk of incident CVD and a two-fold higher risk of CVD-related mortality. Further studies are needed to better understand bisphosphonates’ effect on the cardiovascular system in CKD. 

## Authors’ contributions 

Research idea and study design: DIJ, AJJ; data acquisition: DIJ, AEO; data analysis/interpretation: KAB, DIJ, AJJ; statistical analysis: PTE; reviewer: MLS, SG, AASR; supervision or mentorship: DIJ, AJJ. KAB takes responsibility that this study has been reported honestly, accurately and transparently, and accepts accountability for the overall work by ensuring that questions pertaining to the accuracy or integrity of any portion of the work are appropriately investigated and resolved. 

## Funding 

This study was supported in part by The University of Iowa Clinical and Translational Science Award – NIH (UL1TR002537). Dr. Jalal is supported by R01HL134738 Dr. Jovanovich by VA Merit Award I01CX001985. 

## Conflict of interest 

We have no conflict of interest to disclose. 

**Table 1.** Demographics.

	CKD N = 284		No CKD N = 2,309	
	Bisphosphonate use N = 31	No bisphosphonate use N = 253	Bisphosphonate use N = 286	No bisphosphonate use N = 2,023
Age, years	76 ± 7	76 ± 7	69 ± 9	66 ± 9
Sex, male	3 (10)	106 (42)	28 (10)	1,009 (50)
BMI, kg/m^2^	28 ± 5	30 ± 6	25 ± 5	29 ± 5
Tobacco use, current	2 (6)	12 (5)	18 (6)	188 (9)
Diabetes	4 (14)	71 (31)	20 (7)	320 (16)
Hypertension	18 (58)	199 (79)	120 (42)	1015 (50)
Cardiovascular disease	9 (29)	96 (38)	24 (8)	336 (17)
eGFR, mL/min/1.73m^2^	51 ± 8	50 ± 9	87 ± 14	87 ± 14
Total cholesterol, mg/dL	188 ± 40	172 ± 39	198 ± 34	185 ± 37
HDL, mg/dL	57 ± 15	51 ± 15	69 ± 19	56 ± 17
History of fracture	23 (88)	125 (63)	167 (63)	827 (45)
Malignancy	11 (35)	83 (33)	58 (20)	446 (22)
Steroid use	3 (10)	13 (5)	7 (2)	37 (2)
Coronary Artery Calcium Score	284 ± 502	680 ± 1179	249 ± 501	339 ± 675
Parathyroid hormone (pg/mL)	64 ± 50	58 ± 34	44 ± 17	47 ± 21
Whole-body pelvis BMD, exam 6/7	1.03 ± 0.13	1.12 ± 0.12	1.03 ± 0.1	1.18 ± 0.13
Whole-body spine BMD, exam 6/7	1.03 ± 0.12	1.15 ± 0.15	1.01 ± 0.12	1.19 ± 0.14

Values presented as mean ± SD, number (%). CKD = chronic kidney disease; BMI = body mass index; eGFR = estimated glomerular filtration rate; HDL = high-density lipoprotein; BMD = bone mineral density; SD = standard deviation; chronic kidney disease defined as eGFR < 60 mL/min/1.73m^2^.

**Table 2.** Association of bisphosphonate use with time to outcomes, hazard ratios (95% CI).

Incident cardiovascular disease		
	CKD	No CKD
Event count (full sample)	57/179	253/1949
Unadjusted model	1.72 (0.89 – 3.33)	0.80 (0.55 – 1.18)
Event count (complete cases)	42/133	222/1769
Propensity score-adjusted model	1.66 (0.93 – 2.97)	0.85 (0.63 – 1.16)
Cardiovascular disease-related mortality		
	CKD	No CKD
Event count (full sample)	49/284	93/2309
Unadjusted model	1.33 (0.60 – 2.96)	0.48 (0.21 – 1.10)
Event count (complete cases)	30/201	62/2057
Propensity score-adjusted model	2.20 (1.12 – 4.32)	0.70 (0.36 – 1.37)
All-cause mortality		
	CKD	No CKD
Event count (full sample)	173/284	459/2309
Unadjusted model	1.05 (0.66 – 1.67)	1.14 (0.88 – 1.48)
Event count (complete cases)	104/201	322/2057
Propensity score-adjusted model	1.42 (0.90 – 2.22)	1.59 (1.27 – 1.98)

Propensity score: age, sex, body mass index, current tobacco use, hypertension, diabetes, total cholesterol, high-density lipoprotein, any fracture, malignancy, and steroid use. CKD = chronic kidney disease.
